# Altered Lipid Homeostasis in Sertoli Cells Stressed by Mild Hyperthermia

**DOI:** 10.1371/journal.pone.0091127

**Published:** 2014-04-01

**Authors:** Ana S. Vallés, Marta I. Aveldaño, Natalia E. Furland

**Affiliations:** Instituto de Investigaciones Bioquímicas de Bahía Blanca, Consejo Nacional de Investigaciones Científicas y Técnicas (CONICET) y Universidad Nacional del Sur (UNS), Bahía Blanca, Argentina; University Hospital of Münster, Germany

## Abstract

Spermatogenesis is known to be vulnerable to temperature. Exposures of rat testis to moderate hyperthermia result in loss of germ cells with survival of Sertoli cells (SC). Because SC provide structural and metabolic support to germ cells, our aim was to test the hypothesis that these exposures affect SC functions, thus contributing to germ cell damage. *In vivo*, regularly repeated exposures (one of 15 min per day, once a day during 5 days) of rat testes to 43°C led to accumulation of neutral lipids. This SC-specific lipid function took 1–2 weeks after the last of these exposures to be maximal. In cultured SC, similar daily exposures for 15 min to 43°C resulted in significant increase in triacylglycerol levels and accumulation of lipid droplets. After incubations with [^3^H]arachidonate, the labeling of cardiolipin decreased more than that of other lipid classes. Another specifically mitochondrial lipid metabolic function, fatty acid oxidation, also declined. These lipid changes suggested that temperature affects SC mitochondrial physiology, which was confirmed by significantly increased degrees of membrane depolarization and ROS production. This concurred with reduced expression of two SC-specific proteins, transferrin, and Wilms' Tumor 1 protein, markers of SC secretion and differentiation functions, respectively, and with an intense SC cytoskeletal perturbation, evident by loss of microtubule network (α-tubulin) and microfilament (*f*-actin) organization. Albeit temporary and potentially reversible, hyperthermia-induced SC structural and metabolic alterations may be long-lasting and/or extensive enough to respond for the decreased survival of the germ cells they normally foster.

## Introduction

The first evidence that a testicular temperature a few degrees below that of bodily temperature is essential for normal spermatogenesis was provided almost 90 years ago [1]. Mild testicular heating temporarily suppresses spermatogenesis in several mammalian species [2]. Cellular changes after transient warming are marked by loss of germinal cells at specific developmental stages via apoptotic pathways [3] [4] [5] [6]. This apoptotic process has been considered the critical cause of the impairment of spermatogenesis and of the subsequent testicular regression induced by heat. The most temperature-vulnerable, apoptosis-prone cell types are pachytene spermatocytes, dividing spermatocytes, and early spermatids [7] [8]. However, some time after heat exposures spermatogenesis may eventually recover, as spermatogonia and somatic Sertoli cells (SC), germ cell precursors and spermatogenesis supporters, respectively, are relatively heat-resistant [7].

Previous work from our laboratory showed that, *in vivo*, the heat-stressed rat testis undergoes an intense depletion of lipids that are exclusive of germ cell membranes, as shown after experimental cryptorchidism [9] and after experimental hyperthermia [10]. In both models, glycerophospholipids (GPL) rich in long-chain polyunsaturated fatty acids (PUFA) like 20:4n-6 and 22:5n-6, and sphingomyelins (SM) rich in very long chain PUFA (VLCPUFA) like 28:4n-6 and 30:5n-6, disappeared as the last mature germ cells abandoned the testis and seminiferous tubules became almost exclusively populated by SC. Also in both cases, as germ cells and their endogenous phospholipids decreased approaching their minimum values, the amount per testis of cholesteryl esters (CE) and ether-linked triglycerides (alkyl- and alkenyl-diacylglycerols, here abbreviated ADG) increased, these neutral lipids accumulating mostly PUFA and VLCPUFA among their fatty acids.

The buildup of CE and ADG was considered to be a manifestation of a physiological function of SC that involves lipid processing [10], as these cells normally display phagocytic [11]–[13] and lipid catabolic activities, as shown by an active acid sphingomyelinase [14], as well as a marked ability to oxidize fatty acids [15]. Two weeks after *in vivo* experimental hyperthermia, induced by locally subjecting rat testes during 15 minutes per day to 43°C for 5 successive days [10], the number of germ cells was at its nadir. This not only coincided with peak levels of CE and ADG in the rat testis, but concurred with a considerable buildup of lipid droplets in SC, in agreement with the finding that these two neutral lipid classes are mostly SC products [16]. The buildup of lipid droplets consequent to heat exposures was elegantly confirmed shortly afterwards in the mouse testis *in vivo*, where the expression of two lipid droplet-specific proteins, PLIN2 and PLIN3, was shown to increase after similarly short but daily repeated exposures to moderate hyperthermia [17].

The direct effects of heat exposure on lipid and fatty acid metabolism of SC and the potential impact of these effects on spermatogenesis are still unknown. The aim of this study was to test the hypothesis that, even if SC do not die after repeated heat exposures, they may become temporarily dysfunctional enough to contribute to the reduced survival of the multitude of germ cells that they structurally and metabolically support. The described antecedents suggested that we could expect shifts in lipid metabolism.

In vivo, relatively early lipid-specific changes in SC after heat exposures are difficult to ascertain in the environment of the seminiferous epithelium, where each SC is surrounded by a cohort of germ cells in different stages of their differentiation, many of which are undergoing heat-induced apoptosis. For this reason, in this study we used TM4 cells in culture to investigate possibly deleterious direct effects of hyperthermia on SC lipid and fatty acid metabolism. It was expected that lipids would be altered in a way that would correlate with evidence of SC functional and/or structural alterations. To this aim, we focused on two proteins that are markers of SC functionality and two cytoskeletal proteins.

The results we present in this study demonstrate that hyperthermia induces significant alterations in SC lipid and fatty acid metabolism and that, within the same temporal frame as these lipid changes, SC-specific functions are disturbed. We propose that, although temporary and potentially reversible, most of these derangements may also occur to SC *in vivo* after heat exposures, this having a negative impact on the survival of the germ cells whose development SC support.

## Materials and Methods

### *In vivo* hyperthermia

Adult 4 months-old Wistar rats bred at the INIBIBB were used. Testicular hyperthermia was induced by immersing the scrotal area of sedated rats for 15 min in a water bath kept at 43°C once a day for 3 or 5 consecutive days. Control rats were subjected to the same daily manipulations, except that the water bath was maintained at 22–23°C [10], [18]. At least within the periods surveyed in this study, the testes from these controls gave the same results as those from rats kept in their normal habitat for the same periods at room temperature (22±2°C).

The animals were sacrificed for testis removal either immediately after the last of each of these treatments, or after returning the animals to their cages and waiting for 7 and 14 days after the last of 5 once-a-day exposures. All proceedings were in accordance with *Principles of Use of Animals and Guide for the Care and Use of Laboratory Animals* (NIH regulation) and were approved by the institutional Ethics Committee at INIBIBB-CONICET. Lipids were extracted, separated into classes and analyzed as previously described [10].

### *In vitro* hyperthermia

The TM4 cell line used in this study was established by Mather in 1980 from primary cultures of SC isolated from 11-to 13-day-old BALB/c mice [19]. They were seeded (1×10^5^ cells/dish) and cultured at 37°C for 5 days in Dulbecco's modified Eagle's medium (DMEM) (Gibco BRL, NY) containing 5% fetal bovine serum and 5% horse serum, Penicillin/Streptomycin (100 µU/ml) in a saturated atmosphere of 5% CO_2_ [19]. The dishes were divided into two groups. To mimic the *in vivo* hyperthermia treatment, the experimental group was removed each day from the incubator, momentarily sealed with paraffin film, placed for 15 min in a 43°C water bath, and then returned to the incubator at 37°C. This procedure was repeated once a day during 5 consecutive days. Cell dishes removed and sealed during 15 min but exposed to 37°C were used as the corresponding controls. After the last exposure to 43°C, both experimental and control cells were subjected to the assays described in this section. In the figures of this study the cells are identified as “37°C and 43°C” to discern the temperatures at which have been previously exposed.

### Lipid droplets

The experimental and control SC cultures were washed thoroughly with M1 buffer (150 mM NaCl, 1 mM CaCl_2_, 1 mM MgCl_2_, and 5 mM KCl in 20 mM HEPES buffer, pH 7.4), fixed for 20 min with 2% paraformaldehyde, and stained for 10 min with Nile Red (Molecular Probes), 1.5 µg/ml in M1 buffer. After rinsing three times with M1, the cells were microscopically examined at 510 nm to visualize lipid droplets.

### [^3^H] arachidonic acid (AA) incorporation

In order to evaluate the long-term consequences of *in vitro* hyperthermia on the label distribution among SC lipid classes, once the described heat exposures were finished, control and experimental cells were incubated at 37°C with [^3^H] AA. The labeled fatty acid (1 µCi, specific activity: 65.9 Ci/mmol, PerkinElmer) was mixed with unlabeled AA (final concentration of 3.3 µM) in the presence of lipid-free BSA (4 mol AA/mol BSA), per ml DMEM. Cells were incubated with this medium for 60 min at 37°C to allow for [^3^H] AA incorporation. At the end of this hour, the medium was removed and the cells were washed three times with M1 buffer. After the third washing, some dishes were taken as the initial time (1 hour) control and experimental samples. The rest of the dishes were kept in culture for three days (72 hours) at 37°C with no further interventions. All cells were subjected to lipid extractions in mixtures of chloroform and methanol [20]. Media and washings from control and experimental cell cultures were combined and reserved for assessment of fatty acid metabolization to water-soluble products, in this case labeled with [^3^H].

### Estimation of [^3^H] AA metabolism products

The aqueous phases after the described incubations was mixed with BSA and perchloric acid (2% and 0.5% of the final volume, respectively) to precipitate long-chain fatty acids. The samples were thoroughly mixed and centrifuged for 5 min at 5,000 rpm. This was followed by another round of BSA-mediated fatty acid precipitation. Water-soluble products were determined by measuring the radioactivity in aliquots of the combined supernatants [21].

### Lipid and fatty acid analysis

Lipids were extracted from testes and cultured cells in mixtures of chloroform-methanol [20]. During all preparative procedures lipids were kept under an N_2_ atmosphere. Aliquots of lipid extracts were taken for total lipid phosphorus determination. For lipid class separation, cell lipid extracts were subjected to thin layer chromatography (TLC) along with commercial standards and located under UV light after spraying with diclorofluorescein. Neutral lipids were resolved with n-hexane/diethyl ether (80∶20 v/v), recovering the total polar lipid fraction from the origin of these plates. From this fraction, total GPL and SM were obtained [10]. Appropriate fatty acid methyl esters were added as internal standards for quantification and the separated lipid classes were converted to fatty acid methyl esters. The latter were purified and analyzed by gas chromatography as described in previous work [10].

The labeled lipids from cells incubated with [^3^H] AA were similarly separated into classes by TLC, and visualized in this case by exposure of the plates to iodine vapors. To separate phosphatidylglycerol (PG) from ethanolamine glycerophospholipids (EGP), two-dimension TLC with boric-acid impregnated plates of silica gel G [22] was employed. The silica gel spots containing the lipids were scraped off the plates and, after adding water and mixing with a liquid scintillation mixture, radioactivity was determined by liquid scintillation counting.

### Evaluation of SC viability in culture

TM4 cells were seeded (1×10^5^ cells/dish) at day one, and divided into two experimental groups. As described before, both groups were cultured at 37°C during the following five days, except that the control and experimental groups were removed each day from the incubator and placed during 15 min at 37°C or at 43°C, respectively, in water baths, both being then returned to the same (37°C) culture temperature. At day 5, the metabolic activity of cell mitochondria was estimated by measuring the mitochondrial-dependent conversion of the tetrazolium salt, 3-(4,5-dimethylthiazol-2-yl)-2,5-diphenyltetrazolium bromide (MTT), to a colored formazan product [23]. Absorbance was read at 590 nm on a Jasco spectrophotometer.

The number of dead cells was quantified by propidium iodide (PI) (Sigma Chemical Co, MO) staining. Briefly, PI (2 µM) was added to control and experimental cells and the culture plates were incubated for 30 min at 37°C. The medium was then removed and the cells were washed three times with M1 buffer, followed by observation under a fluorescence microscope at 594 nm to estimate the number of IP- positive cells.

### Evaluation of mitochondrial membrane potential

For localization of actively respiring mitochondria, control and experimental cell cultures were stained for 30 min at 37°C with the red-fluorescent dye MitoTracker Red CMXROS (0.1 µg/ml, Invitrogen). The cells were rinsed three times with M1 buffer, mounted, and examined at 594 nm.

### Cellular oxidant levels and lipid peroxidation

In order to examine total cell production of reactive oxygen substances (ROS), both control and experimental cell culture media were removed and replaced by medium containing the general marker for ROS, 5(6)-carboxy-2′7′-dichlorodihydrofluorescein diacetate (DCDCDHF, Molecular Probes, OR). After 30 min of incubation at 37°C with the probe (10 µM), the medium was removed, the cells were rinsed 3 times with M1 buffer and then imaged with a wide field fluorescence microscope (λex = 538, λem = 590). Lipid peroxidation was measured using the thiobarbituric acid (TBA) assay essentially as previously described [24]. One ml of 30% trichloroacetic acid was added to both control and experimental cell plates (∼60 µg protein), followed by 0.1 ml 5 N HCl and 1 ml of 0.75% TBA, mixed, and transferred to tubes. The latter were heated in a water bath at 100°C for 30 min and centrifuged at 1,000 g for 10 min. The absorbance of TBA reactive substances (TBARS) in the supernatants was read at 532 nm.

### Sertoli cell cytoskeletal proteins

Experimental and control cell cultures were fixed to coverslips, permeabilized with 0.01% Triton X-100 for 20 min, incubated with an α-tubulin antibody for 1 hour (Calbiochem®, Merck KGaA, Darmstadt) (1∶100), washed, and exposed for another hour to the high-affinity F-actin antibody labeled with rhodamine-phalloidin (Santa Cruz Biotechnology, CA) (1∶500), in both cases at room temperature. The coverslips were washed and the cells were exposed to the corresponding secondary antibodies for 1 hour at room temperature.

### Electrophoresis and immunoblotting

In order to measure the levels of SC α-tubulin, β-actin, WT1, and transferrin, control and experimental cell cultures were lysed in cold lysis buffer (3 mM KCl, 50 mM Tris-HCl [pH 7.4], 150 mM NaCl, 1 mM EDTA, 1% Tween-20, and 1% NP-40), supplemented with a protease inhibitor cocktail (Sigma Chemical Co, MO). After centrifugation (13,000 g, 20 min), the supernatants were collected and aliquots were taken for total protein concentration. Proteins were resolved by SDS-PAGE on 10% polyacrylamide gels and transferred to a polyvinylidine fluoride membrane (Immobilon P, Millipore, Bedford, MA). After blocking with TBS-T buffer supplemented with 8% BSA, the membranes were incubated with primary antibodies against α-tubulin (Calbiochem) (1∶5000), β-actin (1∶1000), WT1 (1∶200) and transferrin (1∶200) (Santa Cruz Biotechnology, CA) at 4°C overnight. The membranes were then exposed to the corresponding HRP-conjugated secondary antibodies for 1 hour at room temperature. Immunoreactive bands were visualized using the Amersham ECL detection kit and exposure to x-ray-sensitive film.

### Image acquisition

#### Wide Field Fluorescence Microscopy

Images were obtained using a Nikon Eclipse E-600 microscope. Oil immersion objectives, ×40 or ×60 (1.0 or 1.4 numerical aperture, respectively) were used. Images were obtained with a model ST-7 digital charge-coupled device camera, in a thermostatically cooled SBIG Astronomical Instrument (Santa Barbara, CA). The camera was driven by the CCDOPS software package (SBIG Astronomical Instruments, version 5.02). Appropriate dichroic and emission filters were employed to avoid crossover of fluorescence emission.

#### Confocal Microscopy

Images were obtained with a TCS-SP2 confocal microscope (Leica Mikrosysteme Vertrieb GmbH, Wetzlar, Germany) equipped with an acousto-optical beam splitter and a ×63 (1.2 numerical aperture) water immersion objective.

#### Image analysis

Eight-bit or 16-bit TIFF images obtained from each of the above microscopy techniques were exported to a computer and analyzed with the ImageJ software (National Institutes of Health, Bethesda, MD). The average fluorescence intensity over distinct areas of the cell surface was calculated for cells chosen at random for each experimental condition, selected from phase-contrast images. Background images were acquired from areas without cells from the same specimens.

### Statistical analyses

Values are expressed as mean values ± SD, for at least three independent animal or culture preparations. Data analysis was done using the t- student's test, taking P<0.05 as the criterion for significance.

## Results

### *In vivo* effects of hyperthermia on testicular lipids

**Figure 1:**
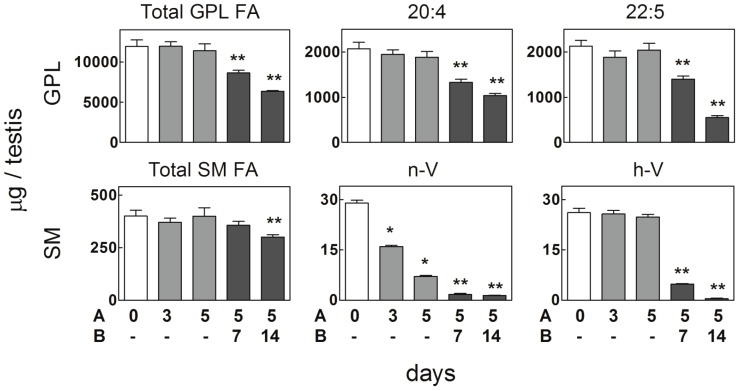
*In vivo* effects of hyperthermia on fatty acid levels of glycerophospholipids (GPL) and sphingomyelins (SM) of rat testis. One single 15°C per day was applied to rat testes for 3 or 5 consecutive days (indicated in A). After the 5^th^ of these 15 min hyperthermia events, the animals were returned to their normal habitat for 7 and 14 days (indicated in B). Samples were obtained from controls (white bars), from animals on the same day of the last hyperthermia treatment (grey bars), and at the end of the described periods with no further treatments (black bars). Asterisks: one (*), points to significant differences with respect to controls; two (**), point to significant differences with respect to day 5.

Three or five exposures of rat testes during 15 min to 43°C, one per day, resulted in no significant changes in the total levels of testicular GPL, as ascertained from their fatty acids (Figure 1). In contrast, as early as after three, and even more markedly after five of these short exposures to 43°C, the disappearance of germ cells was already evident from the lipid standpoint, as a significant decrease took place in the amount per testis of the molecular species of SM that contain nonhydroxy VLCPUFA (n-V) (Figure 1). This particular decrease was consistent with the facts that these species are exclusive components of pachythene spermatocytes [16], and that the latter are the germ cells most vulnerable to temperature [7], [8]. At the same time, the SM species that contain 2-hydroxy VLCPUFA (h-V) remained unaffected affected after these exposures. This observation agrees with the fact that these SM species are components of the most mature and numerous elements of the germ cell line, spermatids and spermatozoa, [16], non-dividing haploid cells that are comparatively more resistant to temperature [7], [8]. Confirming previous results [10], when one and two weeks had elapsed after the last of the 5 consecutive exposures to 43°C, a highly significant depletion of germ-cell-membrane lipids, including PUFA-rich GPL and n-V and h-V containing species of SM, was clearly apparent (Figure 1).

**Figure 2:**
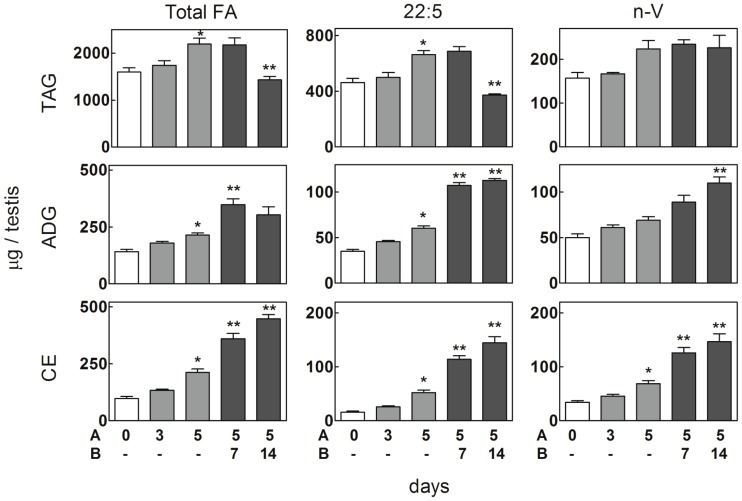
*In vivo* effects of hyperthermia on fatty acid levels of triacylglycerol (TAG), alkyl/alkenyl diacylglycerol (ADG), and cholesteryl ester (CE) in rat testis. As described in Figure 1, one single 15 min exposure per day was applied to rat testes for 3 or 5 consecutive days (indicated in A). After the 5^th^ of these hyperthermia events, the animals were returned to their normal habitat for 7 and 14 days (indicated in B). Samples were obtained from controls (white bars), from animals on the same day of the last hyperthermia treatment (grey bars), and at the end of the described periods (black bars). Other details as in Figure 1.

Regarding three testicular neutral lipids, triacylglycerols (TAG), ADG and CE, neither displayed significant changes in levels after 3 daily brief exposures to 43°C (Figure 2). The three started to show small increases after 5 days of these heat exposures, 22:5n-6 being the fatty acid that contributed the most to their rise. Their levels continued to increase thereafter, reaching maximal values between 1 and 2 weeks later (Figure 2). Most of the original 22:5 rich-TAG of the rat testes are spermatid products [16], [25], but ADG and CE, and particularly their molecular species rich in 22:5n-6 and VLCPUFA, are formed as a result of phagocytic activity in SC. From a lipid standpoint, the fact that this activity was manifested tardily after heat exposures supports our idea that SC were initially affected in their functions because of the heat stress.

### *In vitro* effects of temperature exposures on SC functions

#### Cell viability

**Figure 3:**
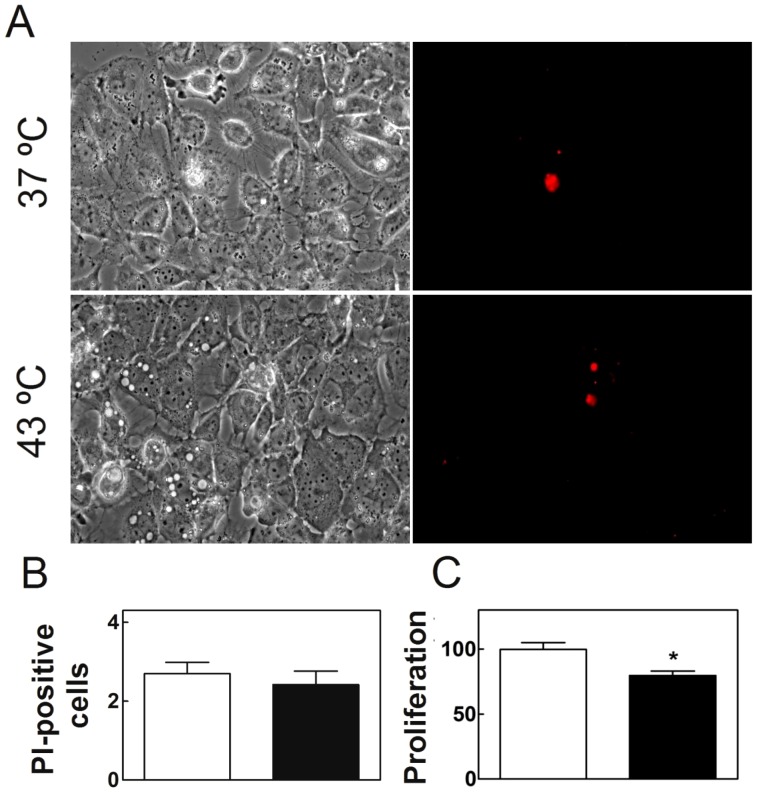
Effects of hyperthermia on SC viability. Cells cultured at 37°C and cells cultured similarly at 37°C but exposed once a day for 15 min to 43°C, both during five days, are compared. A) Phase contrast (left) and fluorescence images (right) of propidium iodide (PI)-positive TM4 cell nuclei. B) Quantification of dead cells after control (white bars) and hyperthermia treatments (black bars), as reported by the labeling with PI (% of dead cells with respect to total cells in each condition). C) Viability of the cells, as evaluated by their capacity to reduce the MTT reagent (% of live cells in each condition) (*p<0.05).

When SC cultured at 37°C were subjected once a day to 15 min hypertermia (43°C) for 5 successive days, on the 5^th^ day the cellular morphology showed no signs of increased apoptosis (e.g. cytoplasmic shrinkage or membrane blebbing) with respect to controls (Figure 3A). This was in agreement with the finding (Figure 3B) that the propidium iodide (PI) assay showed no differences between experimental and control cultures in the proportion of dead with respect to total cells (<2%). The cellular viability, as assessed using the MTT assay, was marginally but significantly reduced with respect to control cells (Figure 3C). Rather than to an increase in cell death, this reduction may be mostly ascribed to a lower rate of cell proliferation of the temperature-stressed cells.

#### Neutral lipid levels and lipid droplets

**Figure 4:**
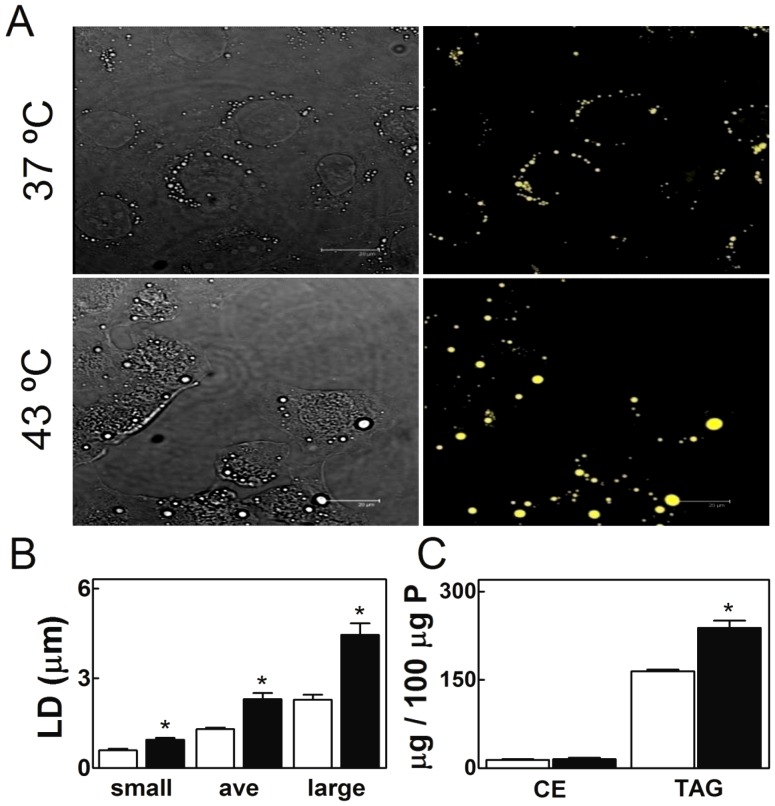
Effects of hyperthermia on SC lipid droplet number and size, and correlation with triacylglycerol (TAG) content. Cells cultured at 37°C and cells cultured similarly but exposed once a day for 15 min to 43°C, both during five days, are compared. A) Phase contrast (left) and fluorescence images (right) of TM4 cells stained with Nile Red to mark lipid droplets. B) Comparison of lipid droplet diameter, and relative abundance of small, average (ave), and large diameter lipid droplets in control (white bars) and experimental (black bars) cells. C) Content of cholesteryl esters (CE) and TAG in these two conditions. (*p<0.05).

Lipid droplets were clearly identified in the cytoplasm of cultured cells by using Nile Red, a lipophilic dye that fluoresces yellow when incorporated in lipid-rich particles. The fluorescence was restricted to neutral lipid inclusions (Figure 4A), and higher fluorescence intensities from lipid droplets of all sizes but especially the largest (Figure 4 B) corresponded to the cells that had been exposed once a day to 15 min hyperthermia for 5 days. A significant increase in TAG levels (Figure 4C) was the most obvious change observed in the lipid composition of SC subjected to these hyperthermia episodes with respect to controls. Other neutral lipids of SC, as was the case of CE, did not show significant increases. Neither TAG nor any of the other lipid classes analyzed, polar or neutral, revealed significant differences in fatty acid composition due to these daily brief exposures to 43°C (data not shown). Thus, in our cultured SC hyperthermia resulted in accumulation of cytoplasmic lipid droplets, whose main component was TAG.

#### Distribution of a labeled fatty acid into lipid classes

**Figure 5:**
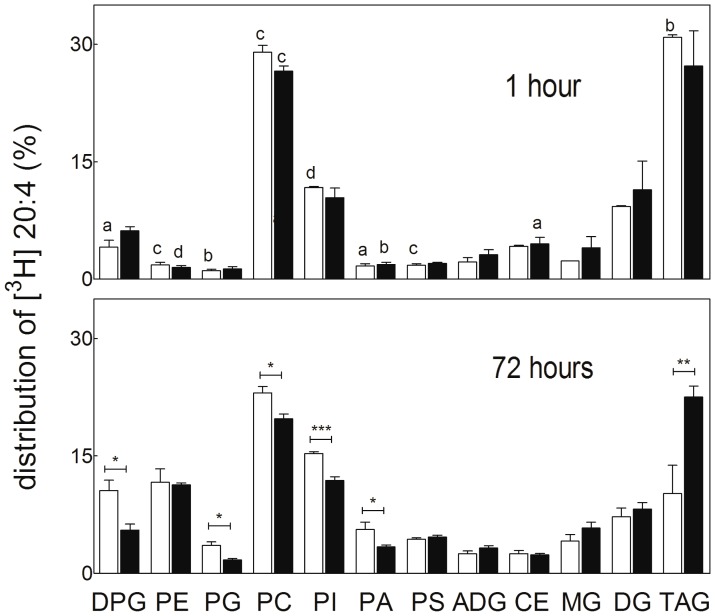
Effects of hyperthermia on the distribution (%) of [^3^H] arachidonic acid (AA) among TM4 cell lipids. Control cells cultured for 5 days at 37°C (white bars) and cells similarly cultured but exposed once a day for 15 min to 43°C (black bars) are compared. After these exposures, the cells were incubated for 1 hour with [^3^H] AA. The medium was removed, the cells were washed, and samples were immediately obtained (indicated as 1 hour) for lipid analysis. The rest of the washed cells were cultured for a further period of 3 days (indicated as 72 hours), in both cases at 37°C, lipids being obtained at the end of this period. Statistically significant differences associated to incubation time (1 hour versus 72 hours) at each temperature condition, are indicated by letters. Differences associated to temperature conditions (previous exposures to 37°C versus 43°C) at each incubation time are indicated with asterisks (*p<0.01; **p<0.008; ***p<0.0005; (a) p<0.030; (b) p<0.008; (c) p<0.005; (d) p<0.0001).

The time-dependent changes in the distribution of [^3^H] AA among lipid classes differed between control cells and cells that had been previously exposed to 5 once-a-day 15 min exposures to hyperthermia (Figure 5). On the 5^th^ day, both were incubated for just 1 hour with the fatty acid, which was then removed and culture continued for 72 hours in order to detect possible long-term effects of hyperthermia. After 1 hour of incubation with [^3^H] AA, TAG was the most highly labeled lipid class in both cases, followed by choline glycerophospholipids (CGP), phosphatidylinositol (PI) and diacylglycerol (DG). Interestingly, significant differences due to temperature exposures became apparent 72 hours later.

In control cells, the percentage of the total label in TAG decreased and that in GPL, notably cardiolipin (DPG), increased significantly between 1 and 72 hours. In cells that had been previously exposed to hyperthermia, the proportion of label in TAG and DPG remained similar between 1 and 72 hours. At this later time (Figure 5), the percentage of label of TAG was about twice higher, while that in DPG was nearly half lower in these cells than in controls.

#### Mitochondrial membrane potential and oxidation products from [^3^H] AA

**Figure 6:**
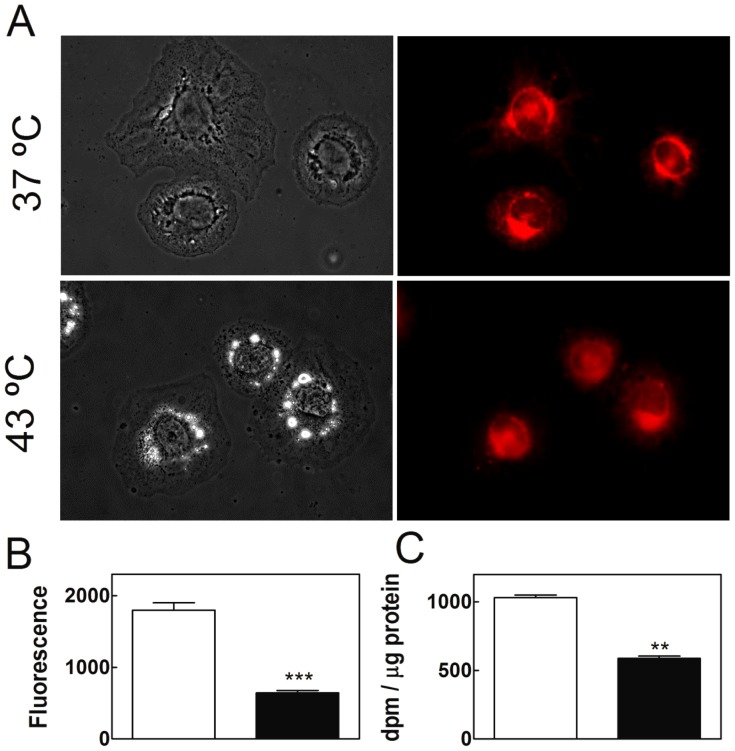
Effects of hyperthermia on mitochondrial functionality. TM4 cells cultured at 37°C and cells cultured similarly but exposed once a day for 15 min to 43°C, both during five days, are compared. A) Phase contrast (left) and fluorescence (right) images of TM4 cells treated with the red-fluorescent dye MitoTracker Red CMXROS. B) Quantification of the fluorescence intensity per cell (expressed on the same basis, as arbitrary units) in control and experimental cells (white and black bars, respectively). C) [^3^H]-labeled aqueous products from cell incubation media from the cells whose lipids had been pre-labeled with [^3^H] AA for 1 hour and then kept in culture for 72 further hours as detailed in Figure 5. (**p<0.002; ***p<0.0001).

Mitochondrial membrane potential (Ψ) is a widely used parameter to monitor the functional status of mitochondria. A useful fluorophore to stain functionally active mitochondria is Mitotracker Red CMXROS. When it enters into an actively respiring cell, this marker becomes oxidized and covalently sequestered in mitochondria. The more polarized the mitochondrial inner membrane (i.e., the greater the Ψ value), the better the uptake of the dye is. The level of CMXROS entry into these organelles is thus directly related to the mitochondrial respiratory activity and inversely related to the extent of inner mitochondrial membrane depolarization. Control cells maintained during 5 days at 37°C exhibited a uniform and intense fluorescence from CMXROS, indicative of actively respiring mitochondria (Figure 6A). In contrast, cells similarly maintained but having undergone 5 once-a-day 15 min exposures to 43°C, showed a significant decrease in such fluorescence (Figure 6A, 6B). This indicates that SC mitochondria were less active, with a reduced Ψ value, confirming that exposure of cells to high temperature induces mitochondrial uncoupling. Figure 6C shows the amount of water-soluble radiolabeled products recovered from the media after 1 and 72 hours of incubation of SC with [^3^H] AA. The cells that had been previously exposed to pulses of 43°C produced, in average, a 40% less of these products than those cultured at 37°C. Thus, the mitochondrial uncoupling that occurred as a consequence of the brief heat exposures reduced significantly the cells ability to oxidize fatty acids.

**Figure 7:**
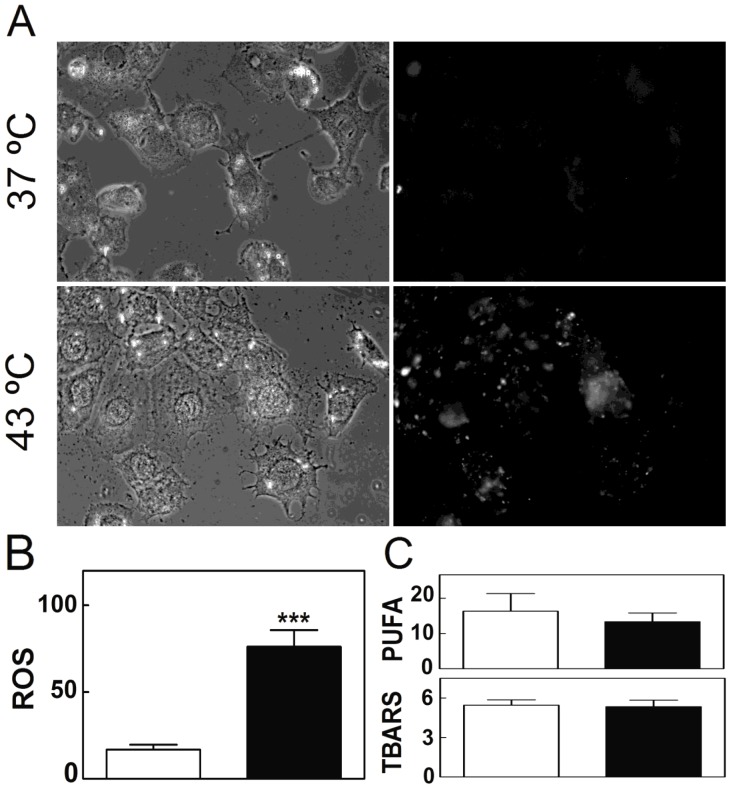
Effects of hyperthermia on SC oxidative stress. Cells cultured for 5 days at 37°C and cells cultured similarly but exposed once a day for 15 min to 43°C, are compared. A) Phase contrast (left) and fluorescence (right) images of TM4 cells treated with the probe DCDCDHF to monitor the oxidative status. B) Reactive oxigen species (ROS), as quantified from the average intensity of the fluorescent emission per cell in control (white bars) and experimental (black bars) conditions. Results are expressed on the same basis, as arbitrary units (*p<0.03; ***p<0.0001). C) Upper panel: percentage of long-chain PUFA in the total lipid of cells; lower panel: absorbance of TBA reactive substances (TBARS), produced by cells exposed to the same conditions.

Another evidence of cell mitochondrial derangement is oxidative stress, characterized by an increase in the levels of reactive oxygen species (ROS). Although ROS can be generated at numerous subcellular sites, the mitochondrial electron transport chain is known to be the major source of intracellular ROS [26]. ROS levels were determined in this study by using 5(6)-carboxy-2′7′-dichlorodihydrofluorescein diacetate (Figure 7A). Once introduced into cells, this probe is deacetylated and easily oxidized in the presence of ROS to 2′,7′-dichlorofluorescein, a highly fluorescent compound. After 5 days of having received the daily episodes of hyperthermia, SC underwent a highly significant increase in ROS levels, in comparison with cell control cultures (Figure 7B). As ROS levels increased, main targets were expected to be the PUFA of cell lipids, besides nucleic acids and proteins. In the present study, despite the increased ROS levels in SC exposed to hyperthermia, no significantly increased levels of TBARS were observed (Figure 7C). This may find an explanation in the fact that the levels of PUFA were similar and notably low, in control and experimental TM4 cells (Figure 7C). These cells, which had been cultured for 72 hours with no replacement of the initial medium, had saturates and monoenoic fatty acids as their main components.

#### Expression of Sertoli cell-specific functional proteins

**Figure 8:**
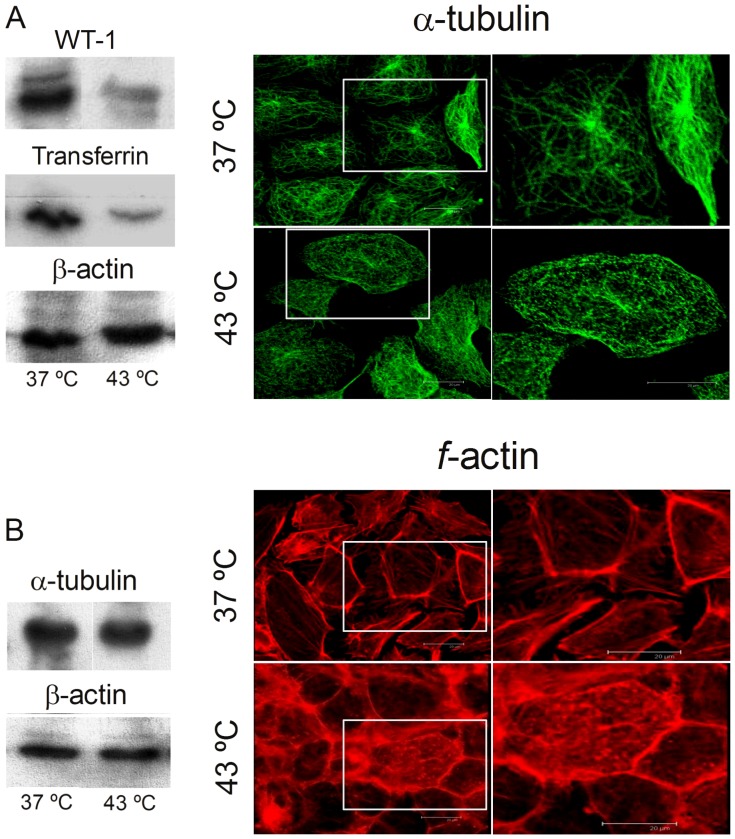
Effects of hyperthermia on specific functional and structural SC proteins. The depicted proteins were obtained from cells cultured for 5 days at 37°C and from cells cultured similarly but exposed once a day for 15 min to 43°C. A) Intensities of the WT1 and transferrin bands, as normalized with respect to the intensity of β-actin. B) β-actin and α-tubulin levels, as normalized with respect to total cell protein. C) Effects of the repeated brief exposures to 43°C, on α-tubulin microtubules and *f*-actin networks, as observed by confocal microscopy. The areas indicated by the white rectangles are shown enlarged on the right panels.

Transferrin is a SC-specific secretion protein that is considered one of the best markers for the evaluation of the SC function [27], and a common marker of SC differentiation [28]. Wilms' Tumor 1 (WT1) protein is a key regulatory factor controlling testicular development [29] that is specifically expressed in SC from early fetal life to advanced adulthood [30]. Taking the amount of β-actin as a reference, the expression of both proteins in SC was significantly decreased after the episodic exposures to hyperthermia (Figure 8A).

#### Sertoli cell cytoskeleton organization

The maintenance of a well-organized cytoskeleton is required for essential cell functions such as division and motion. This organization in turn is highly dependent on an adequate energy supply in the form of ATP, mainly a product of functional mitochondria. To confirm the effects of hyperthermia on SC cytoskeleton-associated proteins, we focused on changes in the organization of tubulin, the largest type of filament that forms cell microtubules, and of *f*-actin, a protein that forms the smallest type of cell filaments (Figure 8, confocal images). In control cells, tubulin microtubules grew out from the centrosome to the plasma membrane, showing a staining pattern characterized by well-defined long tracts extending along the axis of the cells. Similarly, *f*-actin formed networks of uninterrupted filaments throughout the cytoplasm, both proteins thus contributing to the basic structural organization of the SC cytoplasm. In cells that had been subjected to 15 min per day of hyperthermia for 5 days, both, tubulin microtubules and actin filaments were clumped and fragmented. Because the levels of these two proteins, measured by western blot, did not display significant differences due to temperature exposures (Figure 8B), these results can mostly be ascribed to morphological disruption of the SC cytoskeleton.

## Discussion

### Sertoli cells *in vivo*

The long-term consequences on testicular lipids of short (15 min) regularly spaced (one per day) and repeated (a total of 5) episodes of hyperthermia applied *in vivo* to the rat testis have previously been shown to have devastating consequences on germ cells 3–4 weeks after the last of these episodes [10]. At this moment most germ cells had disappeared, for most of the activities regarding lipids that occurred within seminiferus tubules could be attributed to Sertoli cells. This was the case of CE and ADG accumulation, which increased to reach a maximum 1–2 weeks after the last of the repeated heat-stress episodes.

The formation of CE and ADG, as well as part of the TAG, was ascribed in previous work to phagocytic, followed by catabolic activities of SC, the resulting products being temporarily modified to produce neutral lipid intermediates that are temporarily secluded in lipid droplets [10]. Sertoli cell phagocytic activity is physiologically manifested in different stages of spermatogenesis. In the last phases of spermiogenesis, just before condensing, elongating spermatids become nascent spermatozoa, surplus organelles and cytoplasmic components are densely packed in membrane-bound and lipid-rich particles known as “residual bodies”. These bodies are rapidly phagocytized by SC and transiently accumulated in their cytoplasm [31] for further use. In addition, during normal spermatogenesis, more than half the germ cells at various stages of their differentiation naturally die by apoptosis [32]. Sertoli cells in culture rapidly eliminate germ cell-derived apoptotic bodies by phagocytosis and transiently form abundant cytoplasmic lipid droplets [33], the amounts of which decrease 24–48 hours thereafter [15].

These processes were proposed to be involved in the internalization by SC of polar and neutral lipids that derived from apoptotic germ cells that die during harmful conditions such as X-ray irradiation [25] and exposures to hyperthermia [10]. After phagocytosis of germ cell-derived apoptotic and residual bodies, intracellular transport, lipid hydrolysis, and metabolism must naturally follow. Free cholesterol and GPL-derived fatty acids arising from the membranes of such bodies may be combined to produce CE, while GPL-derived fatty acids and diradylglycerols (including diacyl-, akyl-acyl- and alkenyl-acyl- glycerols) may be similarly combined to give rise to the corresponding triradylglycerols. All of these neutral lipids can be temporarily collected in SC in the form of lipid droplets.

In the present study, testicular CE and ADG rich in PUFA and very-long-chain PUFA, markers of SC “doing their job”, required some time to be increased after the repeated testicular exposures to heat. This observation suggests that some functions of SC involved in catabolic processes, e.g. phagocytosis, intracellular lipid transport, or metabolism, were temporarily disturbed by these exposures. This delay sharply contrasted with the results of experimental cryptorchidism [9]. In this case, the increase in neutral lipids levels occurred at much faster rates and then decreased, all in the time frame of a few days. This comparison reinforced our view that spaced but repeated brief exposures to hyperthermia (43°C), albeit not lethal to SC, were deleterious to their *in vivo* functions, by this criterion more damaging to them that a constant exposure to the bodily temperature of 38–39°C typical of cryptorchidism.

Early direct effects of hyperthermia on SC lipids are difficult to evaluate *in vivo* in the intact adult testis because lipid-rich germ cells profusely outnumber them. Conversely, changes in minor lipids in cultured SC are difficult to follow because of the small size of the samples. A further drawback for lipid studies is also the artificial conditions required to maintain cells in culture, such the exposure to air or the requirement for horse and/or calf serum, which provide only a restricted selection of lipids and fatty acids to cells. The species of origin (mouse), the state of maturation (prepuberal) and the transformation(s) undergone by TM4 cells to become cultivable merit some caution when extrapolating to the adult SC situation. Despite these limitations, our *in vitro* model was useful as it displayed most of the biochemical characteristics expected of SC, this allowing the detection of temperature-induced alterations.

### Sertoli cells *in vitro*

Our control and experimental SC cultures were both kept during 5 days at 37°C. The latter differed from the former only in that they were subjected to a single short (15 min) exposure to 43°C every 24 hours. Although these exposures did not significantly affect their survival, SC displayed evidence of altered functions. These included reduced production of SC-specific proteins, mitochondrial dysfunction, propensity of cytoskeleton to collapse, and significant changes in SC lipid homeostasis. One of these changes was directly manifested by an accumulation of TAG in the temperature-exposed SC, demonstrated as an increased concentration of these lipids and a concomitant increase in the amount and size of cytoplasmic lipid droplets. The short but repeated exposures to hyperthermia had consequences on lipid homeostasis that lasted for at least three further days after the last of these exposures, as manifested by a relatively reduced labeling of all major GPL classes with respect to TAG, especially notable for PG and DPG.

**Figure 9:**
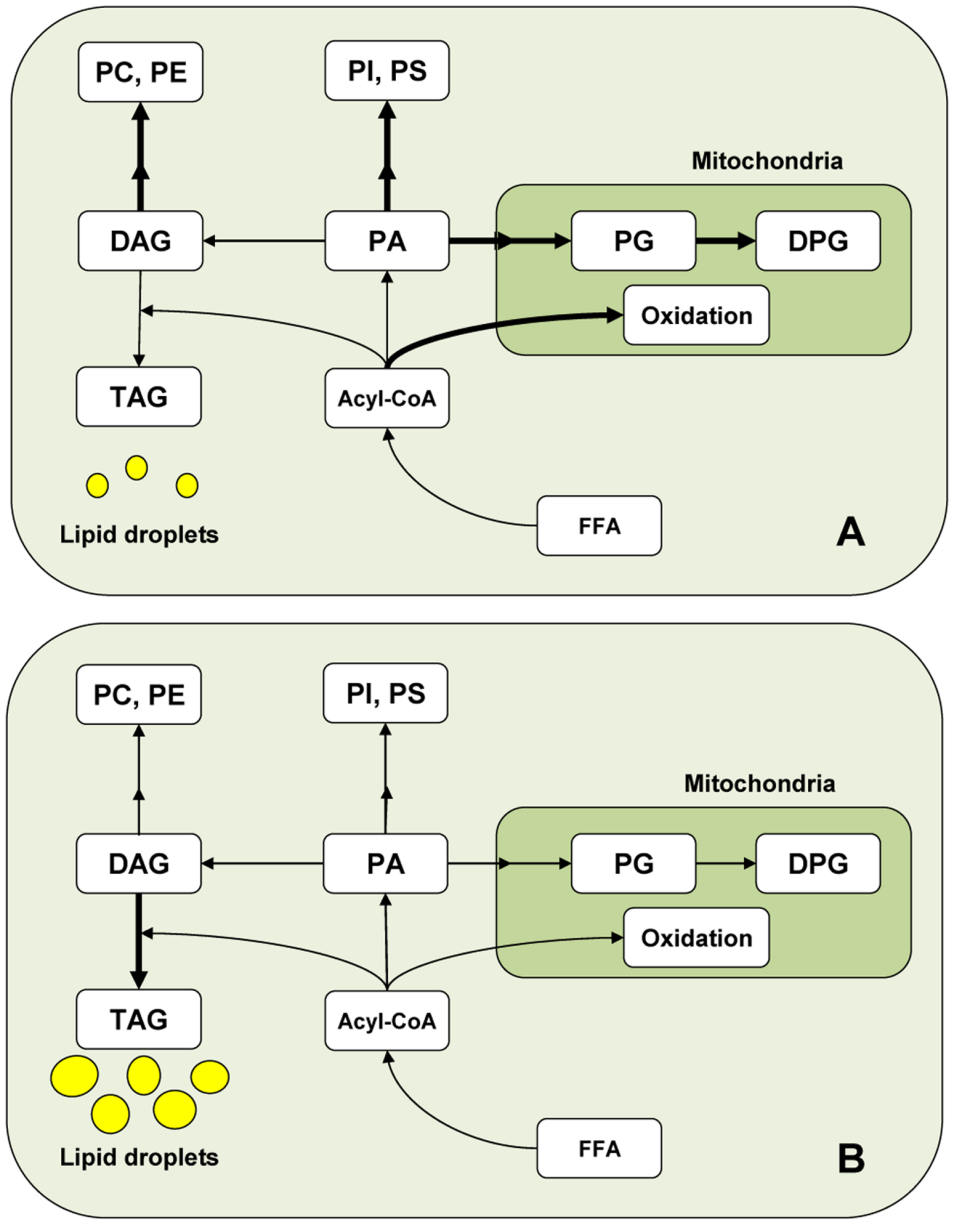
Pathways of lipid labeling, as modified by temperature exposures. A) Cells cultured at 37°C. B) Cells cultured at 37°C but exposed once a day for 15 min to 43°C, in both cases for 5 consecutive days. Double arrows indicate that more than one enzymatic steps and lipid intermediates are involved. The fatty acids that were not used for lipid synthesis and fatty acid oxidation in temperature-stressed cells would be collected in the form of TAG, main components of the increased lipid droplets.

Phosphatidic acid (PA) is known to be located at a branch point in lipid synthesis (Figure 9). It may serve as a precursor of acidic glycerophospholipids (phosphatidylserine, PS; PI; PG; DPG) or may be dephosphorylated into diacylglycerols (DAG). The latter may be normally channeled to the synthesis of PC and PE via the Kennedy pathway on the one hand, or converted into TAG by a DAG-acylCoA acyltransferase (DGAT) on the other (Figure 9A) [34]. In our present model, the part of PA that was not used to synthesize cell GPL after hyperthermia exposures, including mitochondrial ones, could in part have been converted to DAG. These DAG could in turn have functioned as acceptors of the extra fatty acids that were not used for membrane lipid biosynthetic purposes, this contributing to an increased formation of TAG (Figure 9B).

As mitochondrial functions were evidently distressed, unused fatty acids were in part represented by those observed in this study not to be oxidized in heat-stressed with respect to control cells. A part of this surplus of fatty acids may have also been collected in TAG form, and the latter confined in lipid droplets (Figure 9B). At the mRNA level, SC express the *DAGT1* isoform of DGAT, as well as *perilipin 2* [35], a gene encoding for one of the main lipid droplet-associated proteins. The increased formation of TAG and lipid droplets may reflect a defense mechanism of these cells against a potentially harmful accumulation of DAG and free fatty acids. This strategy was apparently successful, as the heat-stressed cells avoided significant death. As DPG synthesis, fatty acid oxidation is an essential mitochondrial function. The fact that these processes were both significantly reduced in temperature-stressed SC indicates, from the lipid point of view, that cell mitochondria were important targets of a SC dysfunctional state caused by temperature exposures.

Regarding specific GPL classes, an unexpectedly active incorporation of [^3^H] AA in the DPG occurred in control SC, suggesting that the biosynthesis of this mitochondrial lipid is a critical metabolic pathway in these cells. The finding that, after heat exposures, the labeling of DPG (and PG, its immediate precursor) was significantly lower than that of control cells, as well as the proportion of label in PA, suggests a derangement of the DPG synthetic pathways (PA→PG→DPG). The mitochondrial inner membrane dysfunction that was concurrently taking place as a consequence of hyperthermia exposures supports this possibility.

The synthesis of the inner mitochondrial membrane lipid DPG requires the import of lipid precursor, PA, which is synthesized in the cell endoplasmic reticulum, into the mitochondria [36], [37]. The temperature-destabilized mitochondria observed in our SC model could have reduced their ability to fulfill such energy-requiring process, resulting in less lipid precursors up to the inner membrane, where the enzymatic steps required to synthesize DPG are located [34], [38].

A decrease in the content of DPG, alterations in its acyl chain composition, and/or peroxidation of its PUFA, have been associated with mitochondrial dysfunction in multiple tissues in a variety of pathological conditions [39]. The importance of DPG in mitochondrial function has suggested that any disturbance affecting the profile of this phospholipid may result in mitochondrial dysfunction, and vice versa [40]. Moreover, DPG has been recently associated with bioenergetic processes [41], [42], and shown to be a critical lipid target of ROS-induced damage [43], [44]. Here we showed that DPG synthesis and mitochondrial functionality in SC were both reduced in the presence of increased ROS levels in cells subjected to heat stress.

*In vivo*, SC are responsible for orchestrating the differentiation of germ cells and for providing the necessary metabolic and structural support for this process. All nutrients or hormonal stimuli to germ cells must pass through SC cytoplasm [45]. In the rat seminiferous epithelium, SC synthesize and secrete binding proteins such as transferrin, one of the hallmarks of SC differentiation [28]. Transferrin is a useful marker for the evaluation of SC function [27] as it decreases in many events that are harmful to SC [46]. The protein that results from the Wilms' tumor gene 1 expression (WT1) is an essential factor for embryogenesis and spermatogenesis [47]. In the testis, SC-specific ablation of *WT1* leads to disruption of inter-cellular protein junctional complexes in developing seminiferous tubules, leading to progressive loss of germ cells [29]. In agreement with previous work in primary cultures of monkey SC [48], our TM4 cells expressed this protein, and reduced such expression after exposures to 43°C.

The finding that transferrin and WT1 protein levels were significantly reduced after five brief and well separated exposures to moderate hyperthermia supports our conclusion that this condition affects SC functions. The disarrangement of the SC cytoskeleton, especially the disruption of SC microtubules, could in part respond for the decreased SC secretory functions.

Oxidative stress is inseparably linked to mitochondrial dysfunction, as mitochondria are both generators of, and targets for, reactive oxygen species. ROS production is known to lead to oxidative damage of mitochondrial membrane components, including lipid PUFA, proteins, and DNA, thereby impairing the ability of mitochondria to synthesize ATP and to carry out a wide range of their metabolic functions, one of which is fatty acid β-oxidation [49]. Mitochondrial rather than cytosolic ROS levels have been pointed out as responsible for decreases in fatty acid β-oxidation [50]. The vulnerability of SC mitochondria to hyperthermia, confirmed in this study by significantly decreased membrane potential values and increased ROS production, agree with the fact that mitochondrial inner membrane functions including ATP generation [51], [52] are deranged by supraphysiological temperatures.

Because the cytoskeleton integrity is involved in the metabolic regulation of mitochondrial respiration and energy fluxes [53], disturbance in the arrangement of microtubules and/or microfilaments may be expected to result in altered mitochondrial functions. This correlation occurred in our *in vitro* model of hyperthermia, as brief but repeated exposures of cells to 43°C concomitantly disrupted selected mitochondrial functions and cytoskeletal organization.

## Concluding Remarks

The adverse effects of temperature on SC functions shown in this study to occur in cultured SC after relatively brief and widely spaced exposures to moderate hyperthermia may be representative of what occurs *in vivo*. A SC cytoskeleton in good shape plays key roles in the maintenance of normal spermatogenesis, as it is involved in the anchoring, translocation and guide of germ cells through the multiple and admirably orchestrated stages of their differentiation. Cytoskeletal functions involve intracellular transport of vesicles and particles, including phagocytized lipid-rich apoptotic and residual bodies released from developing germ cells, displacement on the SC luminal surface of maturing spermatids as the latter become ready to be released as nascent spermatozoa to the seminiferous tubule lumen, and secretion of the luminal fluid required for sperm propulsion out of the testis. All of these functions rely on an adequate supply of ATP, which may be decreased by mitochondrial dysfunction of any cause, including temporary exposures to hyperthermia. That mitochondrial functions are temperature-disturbed in SC is revealed in this study by different criteria, comprising modified lipid homeostasis, reduced levels of fatty acid oxidation and DPG biosynthesis. Because normal spermatogenesis is strictly dependent upon optimal SC functions, these changes could be expected to eventually compromise the role of these cells as spermatogenesis supporters, thereby contributing to the temperature-associated germ cell apoptosis and temporal cessation of the spermatogenic activity induced by hyperthermia. Once these temperature-induced perturbations are surmounted, SC may be able to resume their normal functions, including phagocytosis followed by catabolism and disposal of germ cell-derived materials, as shown in the rat some time after cessation of hyperthermia exposures. However small and potentially reversible, the additive damaging consequences on SC of regularly spaced but daily repeated exposures to moderate hyperthermia may become irreversible if not interrupted. Exposures of this type are not impossible in the case of men; they may evidently occur in those daily exposed to heat for occupational reasons but also inadvertently in those with hot water bathing habits, for such exposures may be considered as a factor when evaluating reduced male fertility.
